# Healthcare Professional Standards in Pandemic Conditions: The Duty to Obtain Consent to Treatment

**DOI:** 10.1007/s11673-020-10048-1

**Published:** 2020-11-09

**Authors:** Sarah Devaney, Jose Miola, Emma Cave, Craig Purshouse, Rob Heywood

**Affiliations:** 1 Department of Law, School of Social Sciences, The University of Manchester, Oxford Road, Manchester, M13 9PL UK; 2 University of Leeds, Leeds, UK; 3 Durham University, Durham, UK; 4 University of East Anglia, Norwich, UK

**Keywords:** Consent, Healthcare professional standards, Duty of care, Pandemic

## Abstract

In the United Kingdom, the question of how much information is required to be given to patients about the benefits and risks of proposed treatment remains extant. Issues about whether healthcare resources can accommodate extended shared decision-making processes are yet to be resolved. COVID-19 has now stepped into this arena of uncertainty, adding more complexity. U.K. public health responses to the pandemic raise important questions about professional standards regarding how the obtaining and recording of consent might change or be maintained in such emergency conditions, particularly in settings where equipment, medicines, and appropriately trained or specialized staff are in short supply. Such conditions have important implications for the professional capacity and knowledge available to discuss the risks and benefits of and alternatives to proposed treatment with patients. The government’s drive to expedite the recruitment to wards of medical students nearing the end of their studies, as well as inviting retired practitioners back into practice, raises questions about the ability of such healthcare providers to engage fully in shared decision-making.

This article explores whether the legal duty on healthcare practitioners to disclose the material risks of a proposed medical treatment to a patient should be upheld during pandemic conditions or whether the pre-eminence of patient autonomy should be partly sacrificed in such exceptional circumstances. We argue that measures to protect public health and to respect autonomous decision-making are not mutually exclusive and that there are good reasons to maintain professional standards in obtaining consent to treatment even during acute pressures on public health systems.

## The U.K. Legal Position on Risk Disclosure

Strongly influenced by the seminal Australian case of *Rogers v Whitaker* ([1992] 175 CLR 479), the landmark decision in *Montgomery v Lanarkshire Health Board* ([2015] UKSC 11 [87]) reaffirmed that U.K. healthcare practitioners are under a duty to inform patients of the material risks of a proposed treatment and of any reasonable alternative or variant treatments. The Supreme Court rejected the old doctor-centred test for materiality and adopted the test in *Rogers,* defining materiality as existing if

in the circumstances of the particular case, a reasonable person in the patient’s position would be likely to attach significance to the risk, or the doctor is or should reasonably be aware that the particular patient would be likely to attach significance to it. (*Montgomery* [2015] [87])

*Montgomery* was grounded in what the court saw as wider developments in law, professional regulation, and society that emphasized the importance of respecting patient autonomy. In relation to risk disclosure, the court held that the law should treat patients

so far as possible as adults who are capable of understanding that medical treatment is uncertain of success and may involve risks, accepting responsibility for the taking of risks affecting their own lives, and living with the consequences of their choices. (*Montgomery* [2015] [81])

Since *Montgomery*, healthcare professionals and lawyers have been attempting to determine its parameters. It represents as much a general philosophy (that healthcare law must be patient -centred) as a detailed account of the legal rule that it provides. The question of how much information is required to be given to patients about the benefits and risks of proposed treatment remains uncertain, with subsequent court decisions compounding a lack of clarity on the boundaries of the duty (Devaney et al. 2019).

COVID-19 has exacerbated this uncertainty, adding complexity and increasing pressures on resources. In the face of insufficient numbers of appropriately trained or specialized staff to cope with the demands the pandemic would place on the system, the government widened the net of possible healthcare providers in the *Coronavirus Act 2020*.^1^ This has resulted in the recruitment of retired practitioners (BBC News 2020) and students nearing the end of their studies, as well as staff who had moved on to other sectors, and the retraining and relocating of existing providers.

Questions can legitimately be raised about whether these groups of practitioners have sufficient up-to-date knowledge and experience about the material risks posed by proposed treatments to engage fully in shared decision-making with patients. Should patients be expected to forgo their right to risk disclosure under these circumstances, whether in relation to treatment for COVID-19 symptoms or any other condition? We consider this in relation to two aspects of the capacity to discuss the risks and benefits of and alternatives to proposed treatment with patients: first, a lack of the skills required to do so; and second, the extreme conditions under which such attempts might be made.

## Requisite Knowledge

In law, the position is that patients are entitled to have standards of care upheld even by practitioners of relative inexperience. Indeed, it should be noted that much of this is settled law from as long ago as the mid-1980s, when the standard criticism was that the law was too generous to doctors. In *Wilsher v Essex AHA* ([1987] QB 730 (CA)), the majority of the Court of Appeal held that the standard of care is to be measured by the task to be done, not the attributes of the person undertaking the task such as inexperience. Glidewell LJ noted that inexperienced colleagues could and would be expected to seek the advice of more experienced colleagues where necessary. In doing so it is highly likely that they will be found to have discharged their duty of care (*Wilsher* at 774).

More recently, this position was confirmed by the Court of Appeal in *FB v Princess Alexandra Hospital NHS Trust* ([2017] EWCA Civ 334), which held that where a doctor in a particular post fails to exercise the degree of skill required for *the task*, they will breach the standard of care.

The courts therefore see the correct standard to apply as relating to the *act performed* rather than the *actor undertaking it*. Inexperience will not lower the standard and nor will more than average experience elevate it (*FB* at [63]). These principles apply to informed consent as much as they do to diagnostic and treatment decisions. This is rooted in the courts’ view of patient expectations of good quality treatment. But that does not mean that such evaluations are context-free, as we demonstrate below.

## Battle Conditions

If the relative experience of the practitioner is irrelevant to the standard of care patients can expect, will the conditions in which they are being treated affect it? *Wilsher* is again instructive here, as the standard of care to be met in “battle conditions” was considered (*Wilsher* at 749). Mustill LJ held that “[a]n emergency may overburden the available resources, and, if an individual is forced by circumstances to do too many things at once, the fact that he does one of them incorrectly should not lightly be taken as negligence” (*Wilsher* at 749). Again, this approach has received support more recently. In *Mulholland v Medway NHS Foundation Trust* ([2015] EWHC 268 (QB)), a case relating to alleged negligence in a busy accident and emergency department (A&E), it was stated that, “[t]he assessment of breach of duty is not an abstract exercise but one formed within a context” ([90]). The reasonable nurse is one “who operates in a busy A&E which has a procedure which the nurse will follow for streaming and which does not contemplate an exhaustive diagnosis being formed” (*Mulholland* [90]). Similarly, busy A&E doctors:

do not have the luxury of long and mature consideration. They take decisions at short notice in a pressurised environment … the standard of care … must be calibrated in a manner reflecting reality. (*Mulholland* [101])

This also applies in relation to informed consent. The decision in *Montgomery* was in large part based on the General Medical Council’s (GMC) consent guidance (2008, ¶23–25, currently under review), which acknowledges the importance of context in what is and is not feasible to expect of doctors. Put simply, nobody expects that the consent process will be the same for an emergency tracheotomy at the roadside following a road traffic accident as it would be for elective surgery. By parity of reasoning, there may be circumstances in the pandemic that constitute “battle conditions,” where it will not be possible for doctors to meet the *Montgomery* standard of disclosure without risking people’s lives and health.

Ethical and regulatory guidelines published since the emergence of the pandemic also support this position. The British Medical Association reassures doctors that they are unlikely to be criticized for care where their decisions are reasonable in the circumstances (British Medical Association 2020). In its ethical guidance on consent published in the light of COVID-19, the GMC has reiterated that the onus is on doctors to act “reasonably and responsibly” in the given circumstances and that they should ensure patients have the time and support to consider material risks “as far as possible” (General Medical Council 2020). Their joint statement with the chief medical officers of the four nations of the United Kingdom acknowledges the challenging circumstances likely to arise in pandemic circumstances and confirms that professional regulators will take these into account in any dealings with their members (Atherton et al. 2020).

Even if a doctor were to be found to have breached their duty of care in a negligence claim, a claimant would still face the hurdle of establishing that the breach caused a compensable harm. This would require them to prove on the balance of probabilities that had they been made aware of the material risks, they would have decided not to undergo the proposed treatment, a notoriously challenging element to prove. Additionally, in some urgent cases, doctors are entitled to withhold information in order to protect the patient. The Supreme Court in *Montgomery* recognized two such instances: one where there is a necessity because treatment is required but the patient cannot consent due, for example, to unconsciousness and the other where the therapeutic exception applies because disclosure would cause the patient serious harm (*Montgomery* [2015] [88]).

In sum then, a doctor is unlikely to be found negligent in such exceptional circumstances. The level of skill and experience will not, in law, justify a failure to disclose material risks, but extreme working conditions can. Can this be justified ethically? Must autonomy be sacrificed for the greater good?

## Individual v Public Health

It might be thought that at the heart of the issue as to whether professional standards on the obtaining and recording of consent should be lowered in emergency conditions lies a question about whether the pre-eminence of patient autonomy should be upheld or sacrificed to other, more communitarian, principles more typical of public health policy such as solidarity or reciprocity but with which they might seem to clash.

It has been argued (Dawson 2010) that a slavish devotion to the pre-eminence of autonomy in bioethics has crowded out other important values which arise in public health considerations. However, we argue that the framing of autonomy as being in conflict with the principles of public health ethics and law fails to reflect more modern understandings of the wider healthcare benefits of respecting individual choice in healthcare. In fact, both are intended to achieve the same overarching aim. Public health has been said to be a concept which “accounts for positive states of well-being” (Coggon and Gostin 2020, 199), its job being “to improve underlying conditions of and for health” (Coggon and Gostin 2020, 200). Public health law has been described as “those aspects of law, policy, and regulation that advance or place constraints upon the protection and promotion of health (howsoever understood) within, between, and across populations” (Coggon, Syrett, and Viens 2017, 72). We argue that the same goes for respecting autonomy. Indeed, as the GMC (2008, ¶3) notes: “[f]or a relationship between doctor and patient to be effective, it should be a partnership based on openness, trust and good communication.” The non-exclusivity of public health ethics and autonomy is also reflected in ethical guidance published by the Royal College of Physicians, which postulates that actions and decision which are “fair, reciprocal, respectful and equitable” can reflect the incorporation of public health ethics into clinical ethics in the context of the current pandemic (RCP 2020).

We argue that respect for autonomy is not necessarily incompatible with the extensive public health measures currently in place and does not impede the goals of those measures. Although there might be exceptional circumstances where these values clash, the law is nuanced enough to take account of this by making an exception to the *Montgomery* standard of disclosure in cases of “battle conditions.” We are confident that doctors will continue to act in partnership with patients as much as the situation allows, though we also acknowledge that some of the particular challenges inherent in the current situation mean that context is more important than ever and that what is feasible depends on the circumstances at the time.

## Conclusion

The willingness of practitioners at the very beginning and end of their careers to step in to provide care and treatment to patients is laudable. In concert with their colleagues already practicing, some of whom have been retrained or relocated beyond their usual expertise, we can imagine that none of them will do so in the hope or expectation that patients will be harmed by any negligent actions. However, the provision of treatment involves risks. To the extent that they are material, patients are entitled to be informed of these. Maintaining usual professional standards of care in this regard as far as is possible in pandemic conditions will help to maintain trust in the profession and contribute to the aims of healthcare and public health law and policy.

## Footnotes

1 See sections 2–5 and schedules 1–4.

## References

[CR1] Atherton, F., C. Calderwood, M. McBride, C. Whitty, S. Powis, and C. Melville. 2020. *Joint statement: Supporting doctors in the event of a COVID-19 epidemic in the UK*. General Medical Council. March 11. https://www.gmc-uk.org/news/news-archive/supporting-doctors-in-the-event-of-a-covid19-epidemic-in-the-uk. Accessed June 23, 2020.

[CR2] BBC News. 2020. *Coronavirus: Tens of thousands of retired medics asked to return to NHS*. March 20. https://www.bbc.co.uk/news/uk-51969104. Accessed April 27, 2020.

[CR3] British Medical Association. 2020. *COVID-19—ethical issues. A guidance note*. https://www.bma.org.uk/media/2360/bma-covid-19-ethics-guidance-april-2020.pdf. Accessed June 23, 2020.

[CR4] Coggon J, Gostin LO (2020). The two most important questions for ethical public health. Journal of Public Health.

[CR5] Coggon J, Syrett K, Viens AM (2017). *Public health law: Ethics, governance, and regulation*.

[CR6] Dawson A (2010). The future of bioethics: Three dogmas and a cup of hemlock. Bioethics.

[CR7] Devaney S, Purshouse C, Cave E, Heywood R, Miola J, Reinach N (2019). The far-reaching implications of Montgomery for risk disclosure in practice. Journal of Patient Safety and Risk Management.

[CR8] General Medical Council. 2008. *Consent: Patients and doctors making decisions together*. https://www.gmc-uk.org/static/documents/content/Consent_English_0617.pdf.

[CR9] General Medical Council. 2020. *Coronavirus: Your frequently asked questions—decision making and consent*. https://www.gmc-uk.org/ethical-guidance/ethical-hub/covid-19-questions-and-answers#Decision-making-and-consent. Accessed June 23, 2020.

[CR10] Royal College of Physicians. 2020. Ethical dimensions of COVID-19 for frontline staff. file://nask.man.ac.uk/home$/Downloads/Ethical%20guidance%20for%20publication%20V3_final_0%20(2).pdf. Accessed June 23, 2020.

